# Gene–Environment Interactions—What Can These Tell Us about the Relationship between Asthma and Allergy?

**DOI:** 10.3389/fped.2017.00118

**Published:** 2017-05-22

**Authors:** Steve Turner

**Affiliations:** ^1^Child Health, University of Aberdeen, Aberdeen, UK

**Keywords:** asthma, atopy, child, eczema, environment, gene, mechanism

## Abstract

Asthma is a common condition, which is associated with atopy and allergic conditions including hay fever, eczema, and food allergies. Asthma and atopy are both complex conditions where genetic and environmental factors are implicated in causation. Interactions between genetic and environmental factors, likely *via* epigenetic mechanisms, are widely thought to be important in determining the risk for developing asthma and atopy. The nature of the relationship between asthma and atopy is unclear and the answer to the question “does atopy cause asthma?” remains unknown. This review explores the relationship between asthma and atopy from a gene–environment interaction perspective and tackles the question “are similar gene–environment interactions present for asthma and atopy?” The main finding is that gene–environment interactions are described for asthma and atopy in children but these interactions are seldom sought for both asthma and atopy in the same population. In the few instances where a gene–environment interaction is related to both asthma and atopy, there is no consistent evidence that similar interactions are common to asthma and atopy. Many plausible gene–environment interactions for asthma and atopy are yet to be explored. Overall, from the gene–environment interaction perspective, there is absence of evidence to better understand the complex relationship between asthma and atopy.

## Introduction

Childhood asthma is a common condition, which affects approximately 1 million children in the UK (1) and six million children in the US (2). Treatment for asthma is available to treat and prevent symptoms, but at present there is no cure for asthma and, therefore, there is a pressing need to understand why some individuals develop asthma. Approximately 50 years of research has yielded a considerable amount of information, which allows us to understand some aspects of asthma pathogenesis.

Asthma has long been associated with atopy [(3), p. 63–70] and is regarded by some individuals as an atopic condition, i.e., one caused by atopy. Asthma is understood to be a complex condition where genetic and environmental factors both contribute to causation, and twin studies suggest that as much as 70% of asthma causation may be explained by hereditary factors [(4), p. 8–14]. The search for “the” asthma gene was called off many years ago with the realization that asthma is a polygenic conditions where approximately ten genes each make a modest contribution to risk [(5), p. 68–74]. There are several environmental exposures, which are associated with childhood asthma and these include exposure to second hand smoke (SHS), inhaled chemicals, mold, ambient air pollutants, some deficiencies in maternal diet, and respiratory viruses (6). Recent work suggests that the relationship between environmental exposures and asthma may change over time; for example, the relationship between SHS and asthma has become slightly stronger over time, perhaps as children become more susceptible (7). Many non-communicable diseases, such as asthma, have both a genetic predisposition and environmental triggers. The gene–environment relationship is nicely captured in the phrase “genetics loads the gun and the environment pulls the trigger.”

Atopy, defined here as production of Immunoglobulin E specific to a common environmental exposure, is a highly prevalent phenomenon in modern children. Some children who are atopic have no symptoms [(8), p. 580–587] and the prevalence of childhood atopy is hard to detect due to it being clinically silent in some individuals, but is likely to be in excess of 30% in Western populations. The prevalence of atopic conditions such as eczema and hay fever is more easily identified due to the presence of symptoms and is close to 30% in many populations despite the use of self-reported diagnosis captured often by different definitions [(9), p. 733–743; (10), p. e008446]. Twin studies of eczema [(11), p. 535–539] and hay fever [(12), p. 2177–2182] suggest that hereditary factors explain up to 80% of causation of these atopic conditions.

The nature of the relationship between asthma and atopy is unclear. While many children with asthma are also atopic and have eczema, hay fever, or food allergies, there are many more children with atopy than with asthma [(9), p. 733–743; (10), p e008446]. In the largest community study of asthma and atopy in the UK, approximately 50% of 6 year olds with asthma (as evidenced by wheeze) were atopic (as evidenced by skin prick positivity) [(13), p. 974–980], i.e., many young children with asthma symptoms are not atopic. The “atopic march,” where at a population level, the prevalences of food allergy, eczema, asthma, and hay fever peak at increasing ages [(14), p. 99–106], has been cited as evidence to support a causal relationship between atopy and asthma but at an individual level, this “march” is very rarely seen [(15), p. e1001748]. While it is possible that atopy may lie on a causal pathway toward asthma, the reverse may also be true (i.e., asthma may lead to the development of atopy) and a third possibility remains that asthma and atopy are independently caused by some other process. The present review is one in a series, which explores the nature of the relationship between asthma and atopy from a number of perspectives. The focus of this review is to review the relationship between asthma and atopy from the gene–environment perspective. Specifically, the hypothesis tested here is: the same gene–environment interactions are associated with both asthma and atopy.

To test this hypothesis, a 2007 review of gene–environment interactions for asthma [(16), p. 1032–1035] was summarized and the literature published after 2007 describing gene–environment interactions for asthma was reviewed. The literature describing gene–environment interactions for atopy and atopic conditions was also summarized. This was not an exhaustive or systematic review, instead, the aim was to identify a number of gene–environment interactions for asthma and for atopy and determine whether there were any common interactions. Papers were included, which described gene–environment interactions for severity of asthma and atopy. Epigenetic mechanisms are covered elsewhere in this series and the interested reader is referred there.

## Methodological Issues for Gene–Environment Interactions

The study of gene–environment interactions for asthma and atopy in childhood is challenging for a number of reasons, which are discussed more fully elsewhere [(17), p. 1229–1240]. The reader should be aware of the following issues before considering the evidence:

1. Definitions of asthma and atopy differs between studies, which makes comparison challenging. For asthma, definitions include self-reported symptoms, current, or “ever” doctor diagnosed asthma and also objective measurements of respiratory physiology, e.g., FEV_1_. For atopy, definitions might include self-reported current or “ever” eczema, hay fever, and food allergy symptoms, and objective measures such as skin prick reactivity and total IgE.
2. Measuring environmental exposures is a challenge and many different methodologies might be applied to the same exposure. Often, exposure is by subjective report, which is known to be potentially unreliable, e.g., exposure to tobacco smoke.
3. Studies require a large sample size to avoid false positive finding and also to detect small effect sizes. Many studies are underpowered. Publication bias means that relatively small studies where associations are seen are published whereas similar sized studies where no associations are seen are not accepted (or even submitted) for publication.
4. Studies require replication in more than one population to be considered generalizable.
5. How are genetic factor(s) of interest selected and related to which environmental exposure(s)? Searching for plausible interactions between candidate genes and environmental exposures can be justified based on current knowledge but this confines research to what is already known.
6. New analytical approaches are required, which can consider large numbers of single-nucleotide polymorphisms (SNPs) and environmental exposures, often which are measured at different ages in the same individual.

## Gene–Environment Interactions for Asthma

**Figure 1:**
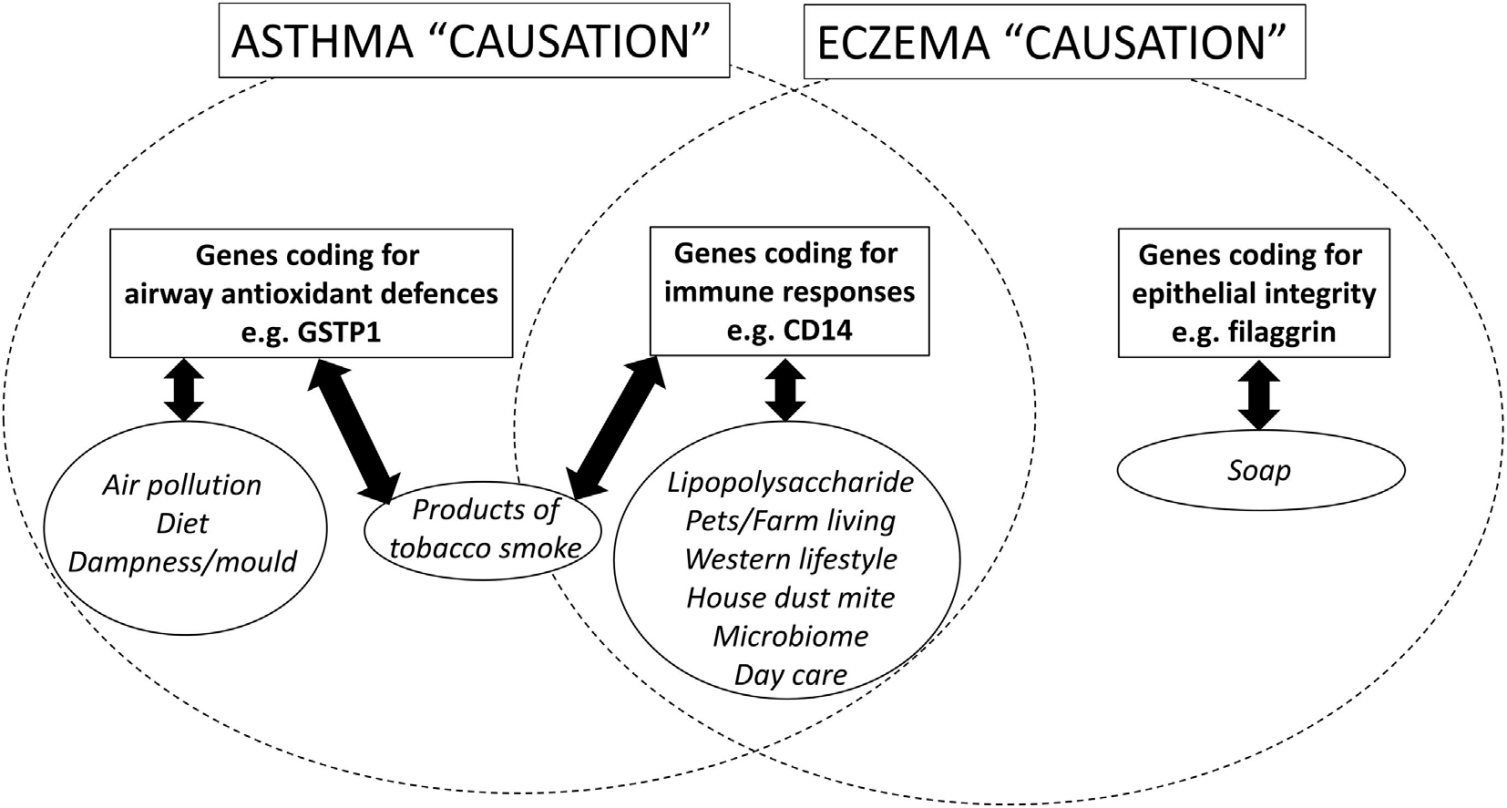
**A schematic diagram, which shows the candidate genetic and environmental factors for asthma and eczema (a surrogate for atopy) in children**.

The 2007 non-systematic review of gene–environment interactions for asthma [(16), p. 1032–1035] found that the majority of the literature had been published since 2000 and was focused in two areas: first, interactions between oxidant exposures (primarily SHS) and variants in genes coding for antioxidant defenses [especially the family of antioxidant enzymes collectively called glutathione-S transferase (GST)]; and second, interactions between exposures to bacteria or bacterial products and variants in genes coding for components of the adaptive and innate immune system (e.g., CD14). See Figure 1.

Since 2007, interactions between oxidant exposures and variants in GST continue to be described, Table 1. Two papers reported on associations between variants coding for the antioxidant protein glutathione S-transferase P1 (GSTP1) and SHS exposure [(18), p. 125; (19), p. 226–232] and neither found an association although one [(18), p. 125] was able to describe increased risk for asthma among those exposed to SHS and low dietary vitamin E, who also were genetically predisposed to oxidant stress. Two other studies related SNPs in the gene coding for GSTP1 and exposure to dampness [(20), p. e30694] and air pollution [(21), p. e52715]; one study described complicated gene–gene and gene–environment interactions for dampness (but not several other exposures) and asthma [(20), p. e30694] while the second described a small increased risk for asthma among those genetically predisposed to oxidant stress and exposed to nitrogen dioxide (NO_2_) [(21), p. e52715]. In addition to variants in genes coding for GST, a study from Hungary also observed a twofold increase in asthma risk for children with rare SNPs, which might reduce host antioxidant defenses but only on exposure to high ambient NO_2_ concentrations [(22), p. 25–33].

**Table 1:** **Summary of examples of where gene–environment interactions for asthma or asthma outcomes have been sought**.

Reference	Genetic variant	Environmental exposure(s)	Outcome reported	Study participants	Association with atopy?	Comments
Brauner et al. [(23), p. 94–97]	17q21 locus (rs7216389)	Antenatal and infant exposure to tobacco smoke and furred pets	Recurrent wheeze (>3 parent reported episodes) at 18 months	101,042 Danish infants	Not reported	Individuals homozygous for the mutant variant and exposed to pets postnatally were at increased risk for recurrent wheeze compared to homozygous wild type [OR 1.6 (1.0, 2.4)]. No interaction was seen for tobacco smoke
Bouzigon et al. [(24), p. 1985–1994]	36 SNPs in 17q21 region	Tobacco smoke exposure in early life (early life not defined)	Doctor diagnosed asthma	1,511 participants in 372 French families, mean age 31 years	No association present for IgE and blood eosinophil counts	Interaction between SNP, early smoking exposure, and early onset asthma
Ramadas et al. [(25), p. 502–508]	3 SNPs in the IL1 receptor antagonist gene	Maternal smoking during pregnancy	Asthma by 10 years	921 participants in a UK birth cohort	Not reported	rs2234678 was associated with a fourfold increased risk for asthma for those whose mothers smoked during pregnancy
Wu et al. [(26), p. 616–622]	6 SNPs in the gene coding for tumor necrosis factor alpha and lymphotoxin A	Tobacco smoke	Asthma and atopy	596 4- to 17-year-old Mexican children	Not replicated for atopy	Two variants in TNF gene (rs1800629 and rs 361525) were associated with increased risk for asthma among non-exposed children
Lee et al. [(18), p.125]	Glutathione S-transferase P1 (GSTP1) (rs1695)	Tobacco smoke and vitamin intake	Doctor diagnosed asthma (ever)	1,124 South Korean children (including 110 with asthma), mean age 9 years	Not reported	Those with low vitamin A intake and exposed to tobacco smoke and homozygous for the GSTP1 mutant variant had increased risk for asthma [OR 4.4 (95% CI 1.6, 12.5)] compared to those with the same genotype but not exposed
Munoz et al. [(19), p. 226–232]	IL-13 (rs20541 and rs1800925), GSTP1 (rs1695), and CPY1A1 (rs1048943)	Tobacco smoke	Asthma	201 children from Mexico	Not reported	No evidence of gene–environment interaction for asthma apparent
Bunyavanich et al. [(27), p. 229–237]	Purinergic receptor (P2Y12) involved in leukotriene cascade. 19 SNPs considered	House dust mite (HDM)	Lung function at 9 years of age (including FEV_1_, BDR and PC_20_)	422 children (mean age 8 years) and 1,266 parents in childhood asthma management program study (USA)	Not reported	5 SNPs tested were associated with lung function and among those exposed to 10 µg/g HDM, homozygous for the rare genotype for 3 of these SNPs were associated with reduced FVC or increased PC_40_ among those exposed to higher HDM concentrations compared to children homozygous for the wild type
Sordillo et al. [(28), p. 885–92.e2]	IL9 SNP (rs2069885) identified in the discovery cohort	HDM	Asthma severity	4 cohorts involved. A discovery cohort, three replication cohorts [including the CAMP study (27), 229–237]	Not reported	Children with mutant variant were at increased risk for asthma attack after exposure to increased HDM (≥10 μg/g dust) in two of the three replication cohorts. The magnitude of effect was approximately a threefold increase
Ege et al. [(29), p. 138; Jan-144]	GWAS (500,000 SNPs) and seven candidate genes	Farm exposure	Doctor diagnosed asthma	1,708 children aged 5–13 years in Germany, Switzerland, Poland, and Austria	Two SNPs previously linked with farm-related exposures were associated with asthma but not atopy	No interactions were found for common SNPs or the seven candidate genes. Not unexpectedly, given the number of SNPs tested, there were interactions with rarer SNPs, which are likely to be false positives
Eder et al. [(30), p. 1117–1124]	7 SNPs in gene coding for Capsase recruitment domain protein (pattern recognition receptor)	Farm exposure	Doctor diagnosed asthma, parent/self-reported wheeze, and hay fever, total IgE	668 children in Germany	Interaction seen for atopy and asthma.	The minor allele of one SNP (rs2075817) as associated with reduced risk for atopy, hay fever, and asthma associated with atopy but only in children living on farms (odds ratio typically fourfold lower)
Su et al. [(20), p. e30694]	GSTP1 (antioxidant gene rs1695), INSIG2 (insulin-related gene), and IL4Ra (rs 1805010)	Indoor dampness	Doctor diagnosed asthma (ever)	1,545 Taiwanese children (including 235 with asthma) mean age 13 years	Not reported	Analysis identified complicated interaction between three of the 17 variants tested and indoor dampness. There were no associations between genetic variants and with other exposures (including antenatal exposure to products of cigarette smoke, pets, cockroach, carpet use)
Hwang et al. [(21), p. e52715]	GSTP1 (rs1695)	Ambient air pollution (averaged over 3 years prior to recruitment)	Doctor diagnosed asthma (ever)	3,825 Taiwanese children (including 295 with asthma) typically aged ≤12 years	Not reported	Same cohort as reference [(20), p. e30694]. Risk of asthma was increased among children homozygous for the mutant variant (val105) per quartile of fine particulates exposure [OR 1.5 (1.0, 2.3)]
Ungvári et al. [(22), p. 25–33]	12 SNPs in gene coding for NFE2L2 (gene product important to antioxidant defenses)	Exposure to high concentrations of NO_2_	Doctor diagnosed asthma	651 children and young adults from Hungary 307 with asthma, mean age of asthmatics 11 years and 22 years for controls	Not reported	Among cases only, rare alleles of 2 SNPs (rs 258882 and rs6721961) were approximately twice as frequent among those with high compared to low exposure

*SNP, single-nucleotide polymorphism; rs, reference SNP identifier number (this gives a unique identifier to each SNP); OR, odds ratio*.

Exposure to products of tobacco smoke is a well-known risk factor for childhood asthma (6) and interaction with genetic variants, which reduce GST have already been discussed, but interactions with variants in other genes might also be important. The ORMDL3 gene is associated with asthma in many populations and is found in the 17q21 region, so not surprisingly, gene–environment interactions have been sought between this area of the genome and exposure to products of tobacco smoke. Two papers [(24), p. 1985–1994; (23), p. 94–97] examined the relationship between many SNPs in the 17q21 region and SHS exposure. One paper found evidence of an interaction between mutant variants and early exposure to SHS and early onset asthma in young adults [(24), p. 1985–1994]. The second paper found no evidence between a single SNP and antenatal exposure to products of tobacco smoke but the mutant variant was associated with a modest increase in risk for early wheeze in association with exposure to pets [(23), p. 94–97]. A third study, from Mexico, reported an unexpected interaction between SNPs in the gene coding for tumor necrosis factor and increased smoking among non-asthmatic children [(26), p. 616–622]. A fourth paper described an interaction between maternal smoking and genetic variant in the IL-1 receptor antagonist for childhood asthma [(25), p. 502–508]. Smoking is an exposure, which is often under reported and which is confounded by many variables including lifestyle and domestic environment, so although not infrequently implicated in gene–environment interactions for asthma, the nature of the relationship cannot be assumed to be causal.

Genetic interactions with house dust mite (HDM) have been sought in two studies [(28), p. 885–92.e2; (27), p. 229–237]; both studies described associations between increased HDM exposure and variants in genes coding for factors associated with the inflammatory response and increased risk for asthma or for respiratory physiological changes associated with asthma. One of these studies was not able to replicate findings in all the populations studied [(28), p. 885–92.e2]. The final exposure considered in gene–environment interactions for asthma in this review is exposure to a farming environment. A study of five populations [(29), p. 138; Jan-144] was not able to replicate interaction between farm exposure and a number of candidate genes for asthma.

## Gene–Environment Interactions for Atopy

At first inspection, there seems to be very little to suggest that there may be common gene–environment interactions for both asthma and atopy. First, genome wide association studies have identified different loci for genes associated with asthma and eczema (a surrogate for atopy). Second, while gene–environment interactions for asthma focus on variants in genes coding for host antioxidant mechanisms, gene–environment interactions for eczema (an atopic condition) are focused on genes associated with epithelial integrity [(31), p. 3–21]. However, GWAS studies [(32), p. 1154–1162] and candidate gene studies [(33), p. 704–714] do find some common areas of the genome and specific variants associated with both asthma and atopy and these regions/genes code for components of the immune system, for CD14, IL4, IL4R, IL13, see Figure 1. Arguably, the most well-recognized gene–environment interaction for atopy is the “endotoxin switch” where individuals carrying with the CC genotype for CD14-159/T (rs 2569190) are at increased risk for atopy at lower endotoxin exposures, but this risk reduces as endotoxin exposure rises [(34), p. 386–392]; those homozygous for CC are at increased risk for non-atopic wheeze at higher endotoxin exposures [(34), p. 386–392].

Compared to gene–environment interactions for asthma, there appear to be considerably fewer publications, which describe gene–environment interactions for atopy; for this review, five papers [(29), p. 138; Jan-144; (35), p. 593–602; (36), p. 231–6.e1–5; (37), p. 621–630; (38), p. 430–437] and one letter [(39), p. 1408–11.e1] were identified, Table 2. All the publications describe associations between variants in genes whose products are part of the adaptive or innate immune system. Variants in the gene coding for CD14 are described in three papers [(35), p. 593–602; (36), p. 231–6.e1–5; (38), p. 430–437] of which two describe interactions with pet exposure [(35), p. 593–602; (38), p. 430–437]. Compared to the literature describing gene–environment interactions for eczema, considered for atopy *per se* is rather sparse. While an interaction between CD14 variants and pets for IgE or eczema is plausible, this need replication in other populations.

**Table 2:** **Summary of examples of where gene–environment interactions for atopy, eczema, hay fever, or food allergy have been sought**.

Reference	Genetic variant	Environmental exposure(s)	Outcome reported	Study participants	Association with asthma?	Comments
Ege et al. [(29), p. 138; Jan-144]	GWAS (500,000 SNPs) and seven candidate genes	Farm exposure	Atopy (type specific IgE > 0.35 kU/L)	1,708 children aged 5–13 years in Germany, Switzerland, Poland, and Austria	One SNP previously linked with farm-related exposures was associated with atopy but not asthma	No interactions were found for common SNPs or the seven candidate genes. Not unexpectedly, given the number of SNPs tested, there were interactions with rarer SNPs, which are likely to be false positives
Bottema et al. [(35), p. 593–602]	IL13 and CD14 (9 SNPs tested)	Tobacco smoke and pet exposure	Total IgE	3,062 children from three cohorts in the Netherlands assessed to age 8 years	Not reported	Minor alleles for 2 CD14 SNPs (rs2569190 and rs2569191) were associated with lower IgE concentrations for those exposed to pets and higher for those not exposed to pets. The magnitude of effect is not stated. Minor alleles for 2 SNPs in IL13 gene were associated with increased IgE concentrations but without interaction
Penders et al. [(36), 231–6.e1-5]	14 SNPs in the toll-like receptor-4 and CD14 genes	Higher burden of stool *E. coli* at 1 month of age	Total IgE- and parent-reported eczema at 2 years	957 children from a Netherlands birth cohort	Not reported	Evidence of gene × gene × environment interaction (TLR4 rs10759932 × CD14 rs2569190 × increased *E. coli* exposure) for elevated IgE
Zhang et al. [(37), p. 621–630]	24 SNPs in 11 immunity-related genes and four IgE response genes	Westernized versus Eastern lifestyle	Current reported eczema and rhinitis	858 children from Finland (“Western”) and Russia (“Eastern”)	No association with asthma and wheeze	SNPs were associated with rhinitis (rs1800896), eczema (rs227306), or elevated IgE (rs324015) among those “exposed” to Western environment. Magnitude of effect not stated
Biagini Myers et al. [(38), p. 430–437]	CD14 (among 7 SNPs tested)	Dog exposure	Diagnosed eczema at two and 3 years of age	762 children from the USA, mean age 3 years	Not reported	Children who were not homozygous for the C variant for −159C/T (rs 2569190) were at reduced risk for eczema [OR 0.4 (0.2, 0.8)] and further reduced if there was a dog in the house [OR 0.4 (0.1, 0.9)]. No associations were seen for exposure to second hand smoke, house dust mite, visible mold
Suzuki et al. [(39), p. 1408–11.e1]	2 SNPs in CD14 gene (rs2569190 and rs5744455) and one in the IL-4 receptor alpha gene (rs1805010)	Day care attendance at ≤2 years of age	IgE	473 children from Japan, mean age 9 years	Not reported	There were interactions between day care attendance and the IL-4Rα variant and also CD14 (rs5744455) for reduced IgE. Children with both the Val/Ile IL-4Rα variant and the CC or CT CD14 variant had the lowest IgE in association with day care attendance

*GWAS, genome-wide association study; SNP, single-nucleotide polymorphism; rs, reference; SNP identifier number (this gives a unique identifier to each SNP); OR, odds ratio*.

## Conclusion

Gene–environment interactions were the “new kids on the block” during the first 10 years of this century, and during this time, there were many publications and regular review articles. Since 2010, there has been a notable reduction in the number of published original research articles and reviews of gene–environment interactions for asthma and atopy (or eczema to be more precise). The shift of focus away from gene–environment interactions may be partly explained by disappointment in the relatively few interactions described and their apparently small effect size. Technological developments may also have shifted scientific thinking and gene–environment-wide interaction studies (GEWIS) may be the “new new kids on the block” [(40), p. 227–230]. An example of a GEWIS is reported in this review [(29), p. 138; Jan-144].

So what is the evidence that there are common gene–environment interactions for asthma and atopy? One paper describing gene–environment interactions for asthma identified in the earlier review [(16), p. 1032–1035] described an interaction between a variant coding for toll-like receptor 2 (rs 4696480) and living on a farm for both asthma and atopy [(30), p. 1117–1124], but this could not be replicated in other populations subsequently [(29), p. 138; Jan-144]. The present review did identify three studies where a relationship between the same gene–environment interaction was sought for both asthma and atopy but no common interaction was found [(24), p. 1985–1994; (26), p. 616–622; (37), p. 621–630]. There is, therefore, *absence of evidence* to robustly answer the question “are the same gene–environment interactions associated with both asthma and atopy?” but there is some *evidence of absence* that the same gene–environment are not related to asthma and atopy.

Studying the relationship between asthma and atopy is not easy due to issues, which include definitions and the coexistence of the two conditions in many individuals in Western populations. The association between asthma and atopy (as evidenced by eczema) may change over time for a population (7), which adds to the challenge in better understanding the nature of the relationship. A paper that is based on epidemiology studies, and which will be frequently cited in this series of reviews, suggests that perhaps as much as 50% of asthma may be attributable to atopy [(41), p. 268–272]. In some countries, many children with asthma are non-atopic [(42), p. 409–416], so the relationship between asthma and atopy is apparently irrelevant for some individuals.

Ultimately, gene–environment interactions are likely to be important to the development of both asthma and atopy but, at this time, do not give a useful insight into the nature of the relationship between asthma and atopy. Other perspectives, for example, intervention studies, may be more helpful in understanding the inter-relationship between asthma and atopy. Some interventions are linked to reduced eczema in preschool children but not asthma [(43), p. 1178–1184; (44), p. 807–813] while other interventions achieve reductions in asthma and eczema into and beyond school age [(45), p. 1046–1051; (46), p. 49–55].

## Author Contributions

The author confirms being the sole contributor of this work and approved it for publication.

## Conflict of Interest Statement

The author declares that the research was conducted in the absence of any commercial or financial relationships that could be construed as a potential conflict of interest.
